# Supplementary material for: Context-dependent enhancer function revealed by targeted inter-TAD relocation
Source: Nat Commun. 2022 Jun 17;13:3488. doi: 10.1038/s41467-022-31241-3 (PMC9205857; doi:10.1038/s41467-022-31241-3)
Supplement: Supplementary file 1 — Supplementary Information [file 41467_2022_31241_MOESM1_ESM.pdf]

Supplementary Information for:

Context-dependent enhancer function revealed by Targeted inter-TAD relocation

Christopher Chase Bolt, Lucille Lopez-Delisle, Aurélie Hintermann, Bénédicte Mascrez, Antonella Rauseo, Guillaume Andrey, and Denis Duboule

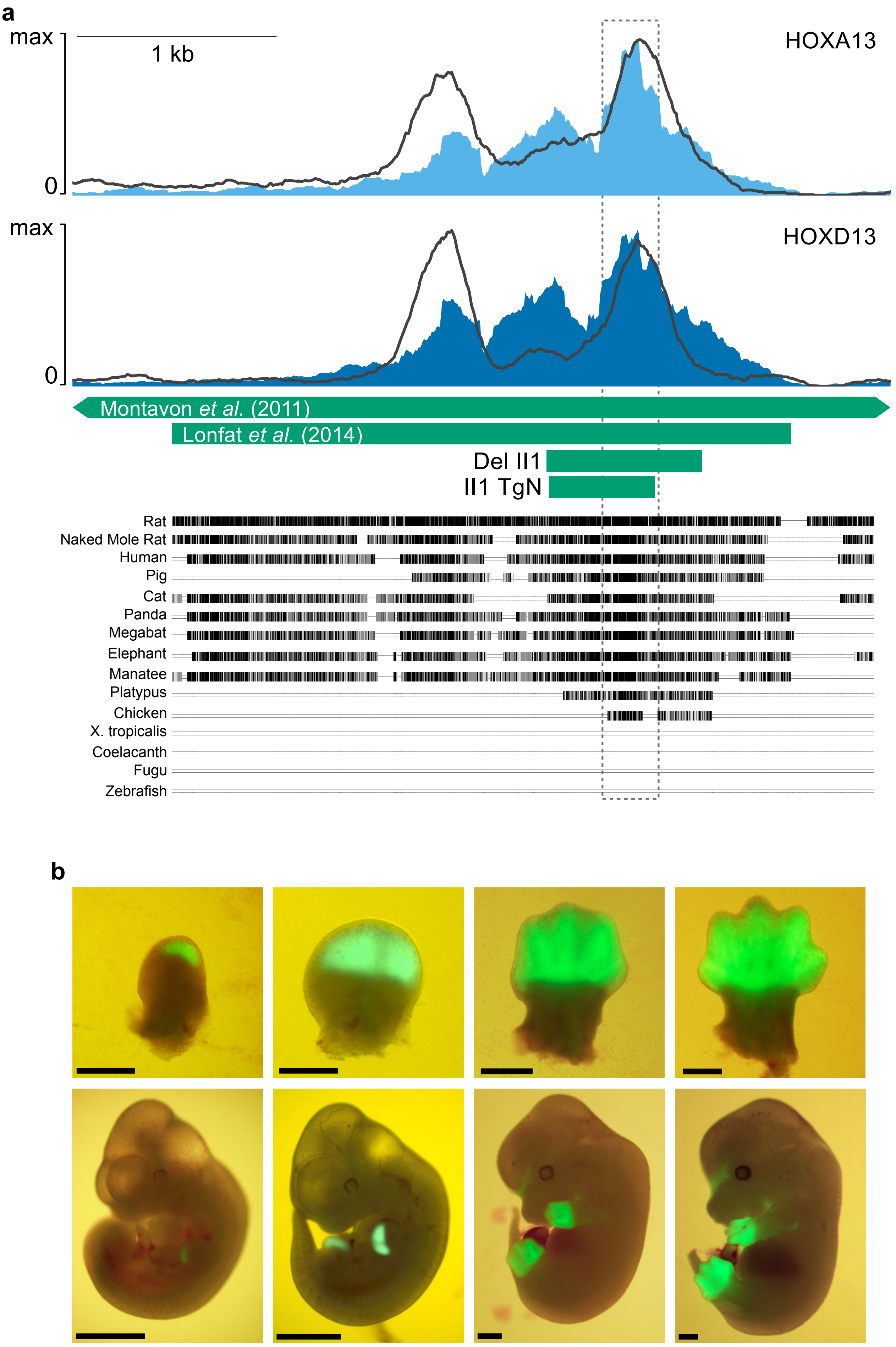

Supplementary Figure 1-1

Supplementary Figure 1-1. **Expression specificity of the distal limb II1 enhancer during limb bud development**. **a**. Magnification of the C-DOM Island II region of the binding profiles of both the HOXA13 (top) and HOXD13 (bottom) transcription factors by CUT&RUN, using E12.5 wildtype distal forelimb cells. The profiles indicated by the dark grey lines are from ChIP-seq using E11.5 whole limb buds [25] and are shown for comparison. The green bars below indicate the regions described as Island II in [17] or in [27]. The Del II1 shows the region deleted in this work (see Supplementary Figure 1-2) and the II1 TgN delineates the transgene used in panel b. Below the genome tracks are the various levels of DNA sequence conservation, showing the presence of the II1 enhancer sequence in amniotes only (vertical dashed rectangle). **b.** Dynamic of the *GFP* fluorescence pattern produced by the II1:*HBB:Gfp* (II1 TgN) enhancer reporter transgene from early day E10 to day E13.5, showing high specificity for distal limb cells and the absence of any detectable staining in proximal cells. The II1 enhancer DNA sequence used in front of the *GFP* reporter system was identical to that used in front of the *LacZ* reporter transgene (Figure 1). Scale bars are 0.5mm.

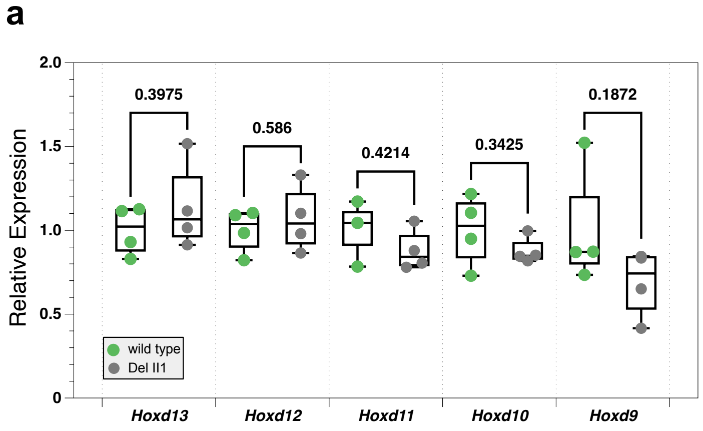

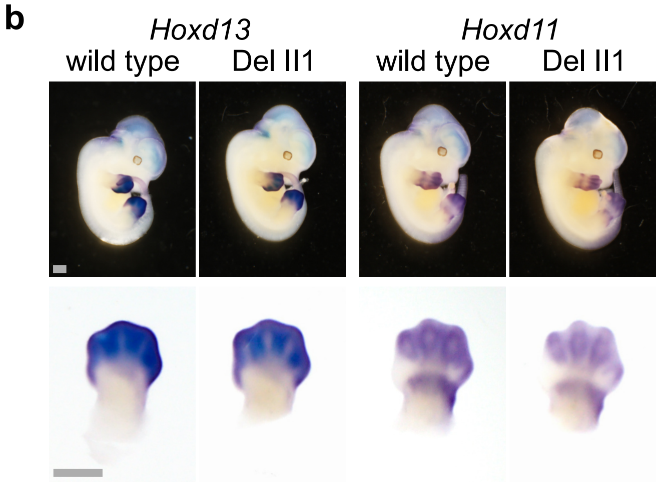

Supplementary FIgure 1-2

Supplementary Figure 1-2. Deletion of the II1 enhancer does not alter *Hoxd* gene expression in the distal limb bud cells. **a**. RT-qPCR for several *Hoxd* genes using distal forelimb bud cells from E12.5 wild type and homozygous Del II1 mutant specimen. Sample size for each genotype was four pairs of distal forelimb tissue. Embryos were from more than one litter. Box plots are interquartile range, whiskers indicate minimum and maximum values. P-values were determined by two tailed Welch's unequal variances t-test. No significant difference in expression was detected for any *Hoxd* genes in the absence of the II1 enhancer. Source data are provided as a Source Data File. **b**. Whole-mount *in situ* hybridizations for *Hoxd13* and *Hoxd11* in embryos homozygous for the II1 enhancer deletion also show no change in their expression domains when this enhancer is deleted from C-DOM. Scale bars are 0.5mm.

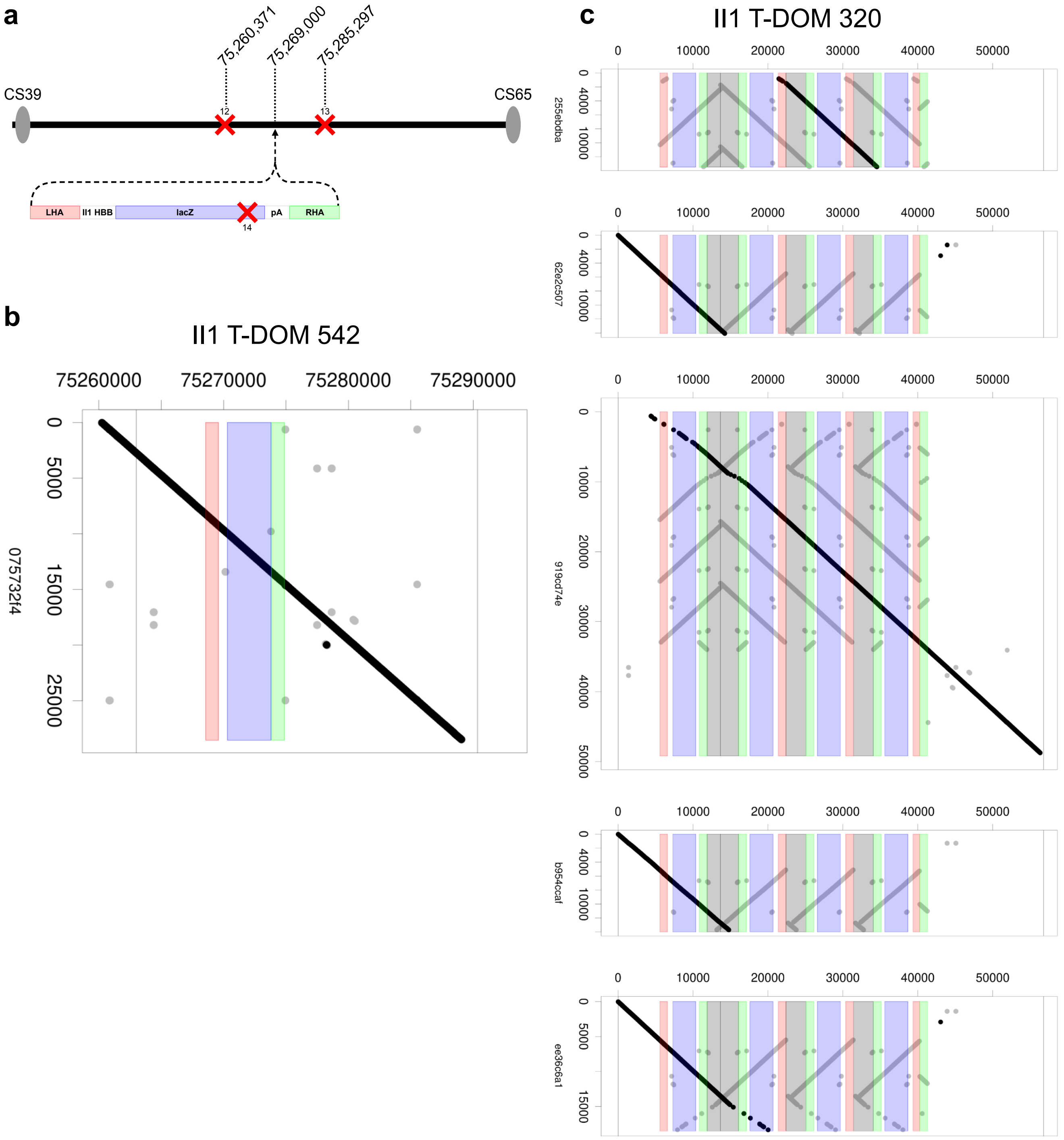

Supplementary Figure 2

Supplementary Figure 2. **Long-read sequencing of the genomic structure after targeted insertion.** **a**. Map of the T-DOM after homologous recombination of the II1:*HBB:LacZ* enhancer-reporter transgene, indicating the location of CRISPR guides used in the nCATS protocol for enrichment of sequencing reads [29]. The red crosses indicate the location of the CRISPR cutting guide (see Supplementary Data 1). Two guides were used outside the II1 transgene (SCS12 and 13) and one guide within the II1 transgene (SCS14). Below the map of the CRISPR cutting position is a map of the transgene construct. The colors indicated for different portions of the construct match to the sequencing alignments below. **b**. Dotplot maps of sequencing reads recovered from the II1 T-DOM 542 allele and showing a clear one-copy recombination at the expected site. The x-axis is the position along the mutant II1 T-DOM 542 chromosome and the values on the y-axis represent the base pair position of the transgene. Each circle represents a 20bp alignment (see methods) so multiple adjacent 20bp matching reads appear as a line. The best matching read is drawn in black, while shorter reads are drawn in grey. **c**. Dotplot maps of five sequencing reads recovered from the II1 T-DOM 320 allele, showing insertion of multiple copies.

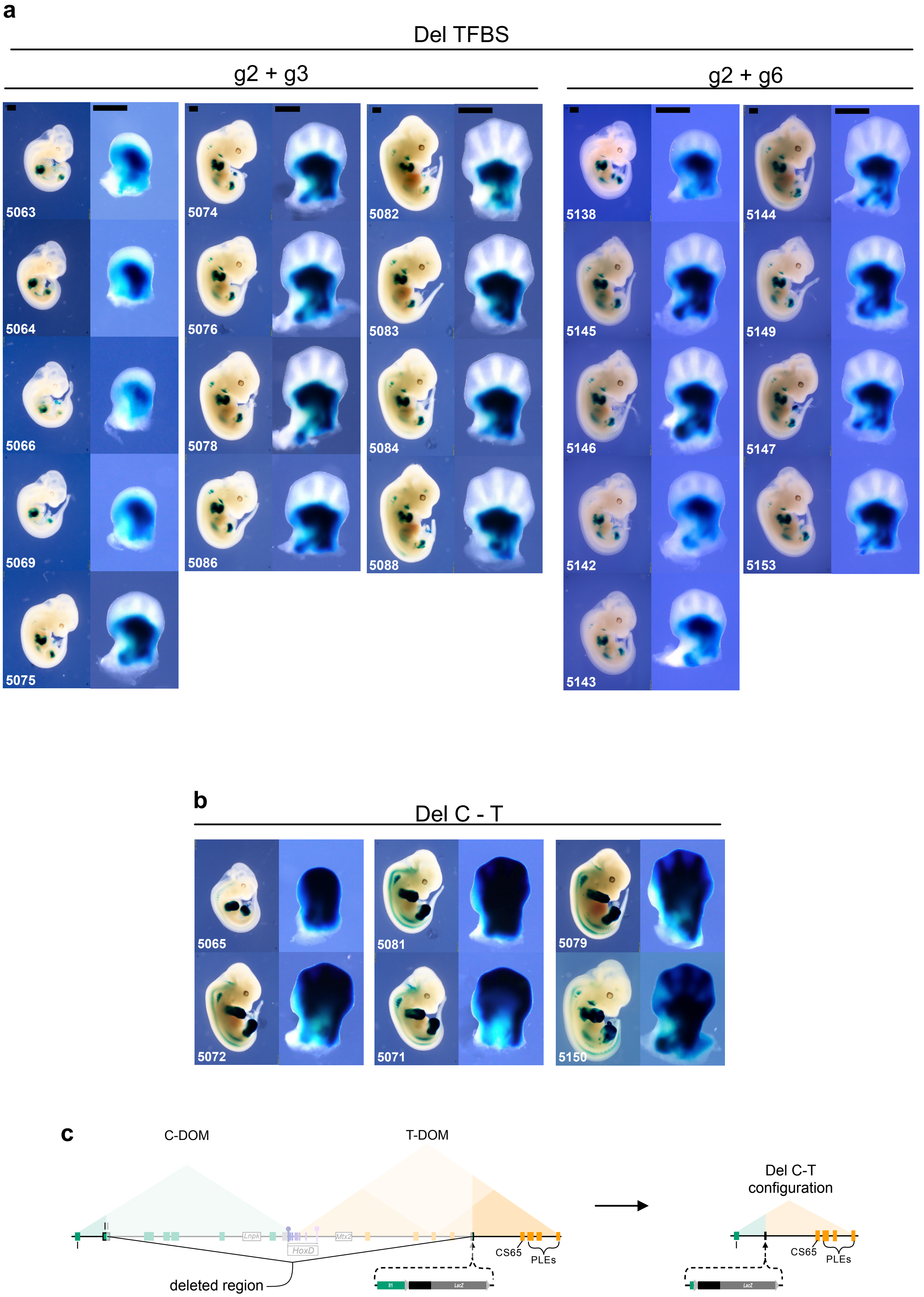

Supplementary Figure 4-1

Supplementary Figure 4-1. **Photos of all embryos containing the deletions of the HOX13 binding sites present in the II1 enhancer-reporter construct targeted into T-DOM**. Individual embryos are numbered. Embryos #5083 and #5146 are already shown in Figure 4b, but are reproduced here for easier comparison. **a**. The g2 + g3 and g2 + g6 deletions removed only binding sites within the II1 enhancer. **b**. Staining of embryos containing the Del C-T deletion. These embryos are positive in both proximal and distal limb bud cells. Embryo #5150 is already shown in Figure 4b but is added here for comparison. **c**. Schematic of the Del C-T deletion. The Del C-T created a large deletion fusing parts of T-DOM and C-DOM due to the presence of the sequence targeted by the guides RNAs on both native (C-DOM) and transgenic (T-DOM). After deletion, the II1 *LacZ* reporter transgene is flanked by a centromeric distal limb enhancer (green) and several telomeric proximal enhancers (CS65, PLEs, orange), thus accounting for its expression in both limb domains. Scale bars are 0.5mm.

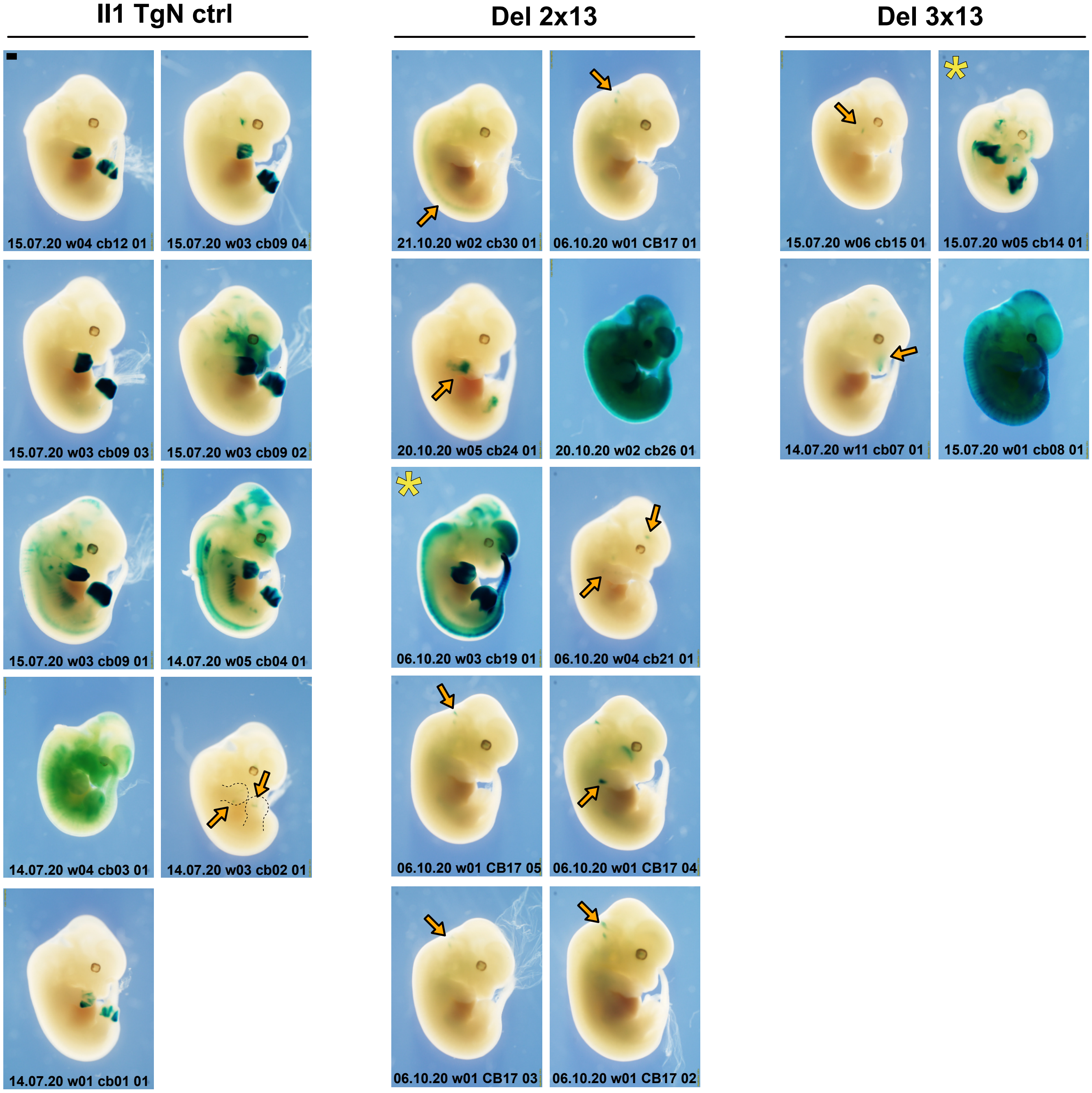

Supplementary Figure 4-2

Supplementary Figure 4-2. **Photos of randomly integrated transgenic embryos stained for *LacZ* expression when HOX13 binding sites were deleted from the II1 enhancer**. The left two columns (II1 TgN ctrl) are control embryos containing the entire II1 enhancer sequence. The two columns in the center (Del 2x13) show stained embryos containing the II1 enhancer element lacking the two centromeric HOX13 binding sites (see Figure 4a, c). The two columns in the right (Del 3x13) show stained embryos lacking all three HOX13 binding sites. The orange arrows indicate the location of *LacZ* staining when it is not detected in the distal limb buds. The unique embryo ID is shown at the bottom of each picture. The three embryos # cb09 04, cb17 05 and cb15 01 are those also displayed in Figure 4d. There are reproduced here for easier comparison. The two outlier embryos showing staining in distal limb cells are shown with a yellow asterisk. Scale bars are 0.5mm.

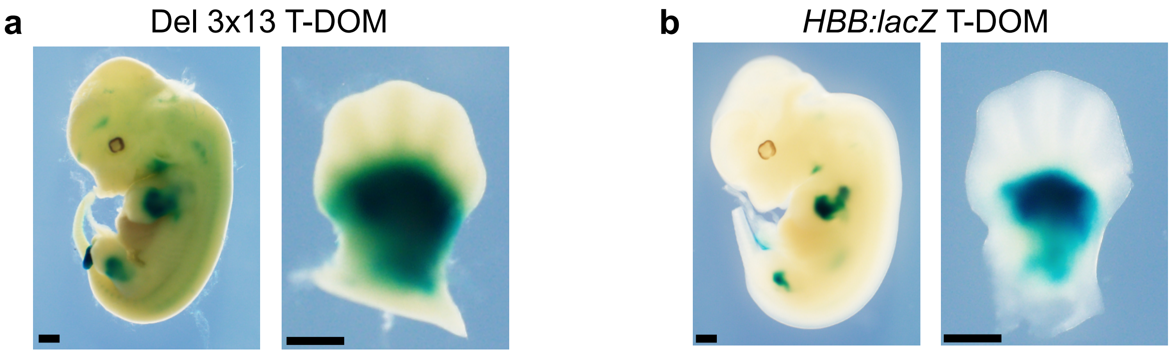

Supplementary Figure 4-3

Supplementary Figure 4-3. *LacZ* staining pattern in control transgenes inserted into T-DOM. a. The *LacZ* staining in a control embryo containing the Del 3x13 variant of the II1 enhancer. This variant contains the same targeting construct as the II1 T-DOM 542 allele, but the II1 enhancer has been replaced with the same variant used to produce the randomly integrated Del 3x13 (Figure 4d). The staining is very strong in the proximal limb and completely absent in the distal limb. This corroborates the observation from Figure 4, that the three HOX13 binding sites in II1 are necessary for the distal limb staining either as a randomly integrated transgene or a targeted insertion transgene in the T-DOM. **b**. The *LacZ* staining in a control embryo that does not contain the II1 enhancer element. This transgene contains the same targeting construct as the II1 T-DOM 542, but without the enhancer. The staining is very strong in the proximal limb and completely absent in the distal limb. Scale bars are 0.5mm.

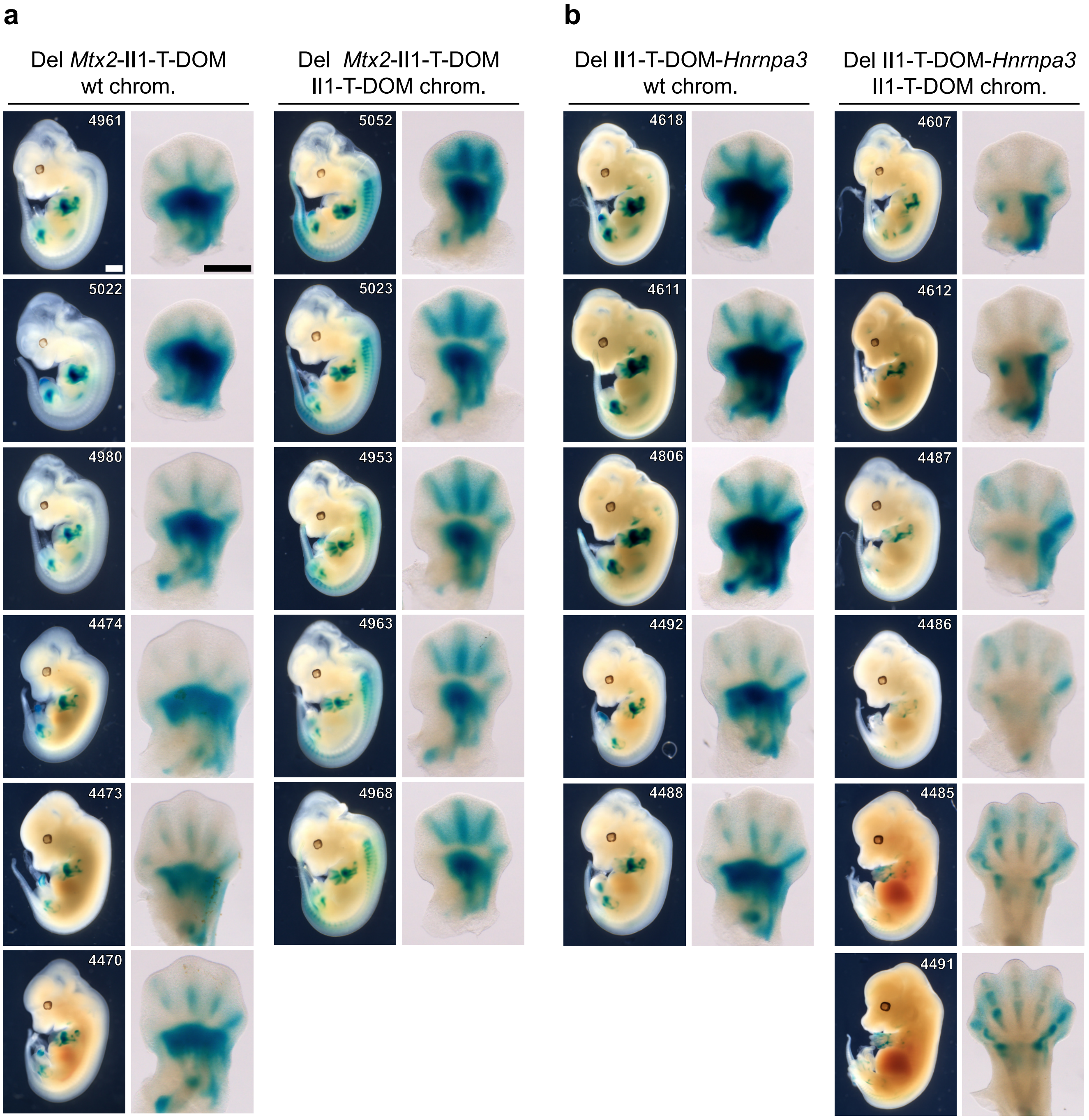

Supplementary Figure 5

Supplementary Figure 5. **Photos of all embryos carrying large deletions flanking the insertion of the of II1 enhancer-reporter construct within T-DOM** (see Figure 5b, c). **a**. Images of all embryos containing the deletion Del *Mtx2*-II1-T-DOM and control embryos (left) from the same litters. **b**. All embryos containing the deletion Del II1-T-DOM-*Hnrnpa3* and control embryos from the same litters (left). All embryos were genotyped for the expected deletion. All embryos with the expected deletions are represented here unless they produce ambiguous PCR results or were mosaic for the deletion. The embryos #4470, 5023, 4492, 4491 from Figure 5 are reproduced here for easier comparison. Scale bars are 0.5mm.

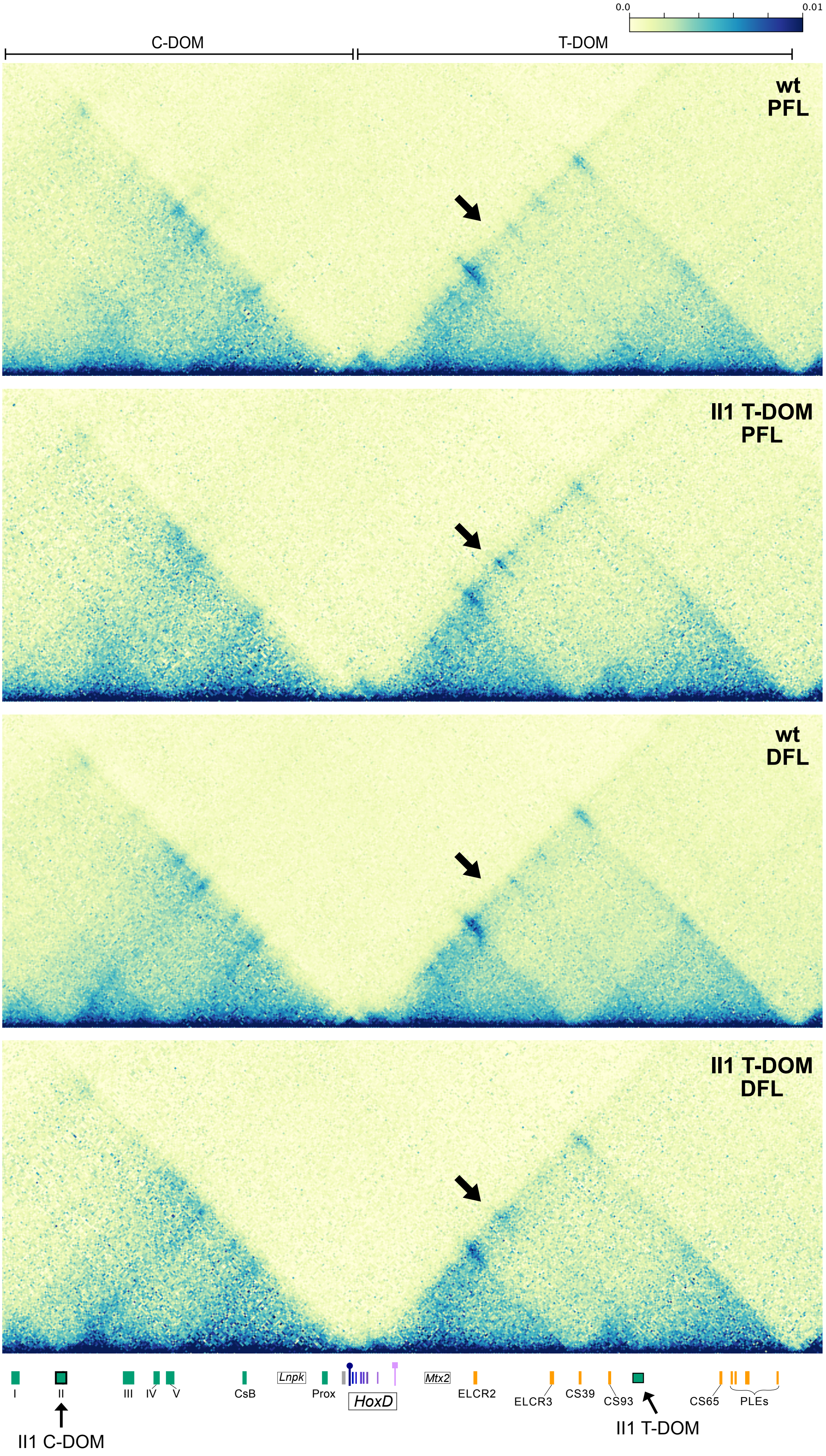

Supplementary Figure 6

Supplementary Figure 6. **Capture Hi-C maps**. Capture Hi-C maps covering the entire *HoxD* locus for wild type (wt) and the II1 enhancer recombined within T-DOM (II1 T-DOM), using both proximal (PFL) and distal (DFL) forelimb cells samples (mm10 chr2:73950000-75655000). The new contacts formed between the II1 enhancer-promoter sequence and the *Hoxd* gene cluster are indicated by the black arrow. The contacts were scored in both PFL and DFL cells, yet with a difference in resolution, being more diffuse in the DFL cells (see Figure 6). The black arrows below points to the location of the II1 enhancer within C-DOM and the transgene integration site within T-DOM. The *HoxD* cluster position is indicated at the center. The *Hoxd13* gene is indicated by a purple pin with a circle and the *Hoxd1* is indicated with a square. Distal limb enhancers are indicated by green boxes, and proximal limb enhancers are indicated by orange boxes. The gene bodies of *Lnpk* and *Mtx2* are indicated by white boxes with black borders.

Supplementary Data 1: Primers for genotyping, CRISPR guides, and relevant DNA sequences.

Supplementary Table 2: Sanger sequencing of mutated configurations in Supplementary Figure 4.
