# Supplementary material for: Context-dependent enhancer function revealed by targeted inter-TAD relocation
Source: Nat Commun. 2022 Jun 17;13:3488. doi: 10.1038/s41467-022-31241-3 (PMC9205857; doi:10.1038/s41467-022-31241-3)
Supplement: Supplementary file 3 — Description of additional Supplementary File [file 41467_2022_31241_MOESM3_ESM.pdf]

Descriptions of Additional Supplementary Data Files

Supplementary Data 1: Primers for genotyping, CRISPR guides, and relevant DNA sequences.

Supplementary Data 2: Sanger sequencing of mutated configurations in Supplementary Figure 4.
