# Supplementary material for: Context-dependent enhancer function revealed by targeted inter-TAD relocation
Source: Nat Commun. 2022 Jun 17;13:3488. doi: 10.1038/s41467-022-31241-3 (PMC9205857; doi:10.1038/s41467-022-31241-3)
Supplement: Supplementary file 6 — Reporting Summary [file 41467_2022_31241_MOESM6_ESM.pdf]

nature portfolio

Corresponding author(s): Christopher Chase Bolt
Denis Duboule

Last updated by author(s): 2022.05.17

Reporting Summary

Nature Portfolio wishes to improve the reproducibility of the work that we publish. This form provides structure for consistency and transparency in reporting. For further information on Nature Portfolio policies, see our Editorial Policies and the Editorial Policy Checklist.

Statistics

For all statistical analyses, confirm that the following items are present in the figure legend, table legend, main text, or Methods section.

n/a	Confirmed	
☐	☒	The exact sample size (*n*) for each experimental group/condition, given as a discrete number and unit of measurement
☐	☒	A statement on whether measurements were taken from distinct samples or whether the same sample was measured repeatedly
☐	☒	The statistical test(s) used AND whether they are one- or two-sided<br>*Only common tests should be described solely by name; describe more complex techniques in the Methods section.*
☐	☒	A description of all covariates tested
☐	☒	A description of any assumptions or corrections, such as tests of normality and adjustment for multiple comparisons
☐	☒	A full description of the statistical parameters including central tendency (e.g. means) or other basic estimates (e.g. regression coefficient) AND variation (e.g. standard deviation) or associated estimates of uncertainty (e.g. confidence intervals)
☐	☒	For null hypothesis testing, the test statistic (e.g. *F*, *t*, *r*) with confidence intervals, effect sizes, degrees of freedom and *P* value noted<br>*Give P values as exact values whenever suitable.*
☒	☐	For Bayesian analysis, information on the choice of priors and Markov chain Monte Carlo settings
☐	☒	For hierarchical and complex designs, identification of the appropriate level for tests and full reporting of outcomes
☒	☐	Estimates of effect sizes (e.g. Cohen's *d*, Pearson's *r*), indicating how they were calculated

Our web collection on statistics for biologists contains articles on many of the points above.

Software and code

Policy information about availability of computer code

Data collection	Olympus cellSens Standard 2.1 was used for image capture of embryos.<br>CHOPCHOP v3 was used to select CRISPR guides for the production of Il1 T-DOM lines 542 and 320.<br>CRISPR guides for all other targeted cutting were selected from the UCSC genome track CRISPR Targets - CRISPR/Cas9 -NGG Targets, whole genome, update 2019-10-14.
Data analysis	DataGraph 4.6.1 was used to generate box plots in Figure S1B and calculate p-values.<br><br>Most of NGS plots were realized with pyGenomeTracks version 3.7<br><br>For ATAC-seq analysis:<br>cutadapt version 1.16 was used to remove the adapters and bad quality bases<br>bowtie2 version 2.4.1 was used to map the reads<br>bamtools version 2.4.1 was used to remove poor mapping quality reads and reads mapping to chrM<br>picard version 2.18.2 was used to remove the PCR duplicates.<br>bedtools version 2.30.0 was used to convert bam to bed<br>macs2 version 2.1.1.20160309 was used to call peaks and generate coverage<br>wigToBigWig version 357 was used to convert coverage from macs2 to bigwig<br><br>For CUT&RUN analysis:<br>cutadapt version 1.16 was used to remove the adapters and bad quality bases<br>bowtie2 version 2.4.1 was used to map the reads<br>samtools version 1.8 was used to remove poor mapping quality reads

picard version 2.18.2 was used to remove the PCR duplicates.
macs2 version 2.1.1.20160309 was used to call peaks and generate coverage
wigToBigWig version 357 was used to convert coverage from macs2 to bigwig

For ChIP analysis:
cutadapt version 1.16 was used to remove the adapters and bad quality bases
bowtie2 version 2.4.1 was used to map the reads
samtools version 1.8 was used to remove poor mapping quality reads
macs2 version 2.1.1.20160309 was used to call peaks and generate coverage
wigToBigWig version 357 was used to convert coverage from macs2 to bigwig

For motif discovery and scan:
Homer version 4.11 was used

For cHi-C analysis:
cutadapt version 1.16 was used to remove the adapters and bad quality bases
HiCUP version 0.6.1 with bowtie2 version 2.2.6, samtools version 1.2 and docutils version 0.13.1 was used to process the fastq.
A custom python script available at https://testtoolshed.g2.bx.psu.edu/repository/download?repository_id=be5040251cd4afb7&changeset_revision=44365a4feb3b&file_type=gz to convert the bam to a text file.
The tool filter version 1.1.1 from galaxy was used to keep good quality pairs with both mates in the capture region.
cooler version 0.7.4 was used generate matrices as balanced cool files.
HiCExplorer version 3.6 was used to compute differences between cool files.

For nCATs/minION analysis:
guppy_basecaller version 5.0.16+b9fcd7b was used for basecalling.
minimap2 version 2.15 was used to map the reads.
samtools version 1.10 was used to filter the reads and select the interesting reads.
bedtools version 2.27.1 was used to convert bam to bed
seqtk version 1.3.0 was used to get the sequence of the interesting reads.
A perl script available at jura.wi.mit.edu/page/papers/Hughes_et_al_2005/tables/dot_plot.pl was used to generate table with matching positions.
R version 4.0.2 was used to generate the dotplot figure.

For manuscripts utilizing custom algorithms or software that are central to the research but not yet described in published literature, software must be made available to editors and reviewers. We strongly encourage code deposition in a community repository (e.g. GitHub). See the Nature Portfolio guidelines for submitting code & software for further information.

Data

Policy information about availability of data

All manuscripts must include a data availability statement. This statement should provide the following information, where applicable:

- Accession codes, unique identifiers, or web links for publicly available datasets
- A description of any restrictions on data availability
- For clinical datasets or third party data, please ensure that the statement adheres to our policy

All raw and processed datasets are available in the Gene Expression Omnibus (GEO) repository under accession number GSE194114. All scripts necessary to reproduce figures from raw data are available at https://github.com/lldelisle/scriptsForBoltEtAl2022.

Field-specific reporting

Please select the one below that is the best fit for your research. If you are not sure, read the appropriate sections before making your selection.

☒ Life sciences ☐ Behavioural & social sciences ☐ Ecological, evolutionary & environmental sciences

For a reference copy of the document with all sections, see nature.com/documents/nr-reporting-summary-flat.pdf

Life sciences study design

All studies must disclose on these points even when the disclosure is negative.

Sample size	For all WISH, at least 3 biological replicates were evaluated (3 embryos). For RTqPCR, 4 embryos of each genotype were used which is a large enough sample size to estimate expression variance determined by genotype and stage and other biological differences. Each gene was evaluate in three technical replicates for biological replicate which is sufficient to accommodate pipetting error. CUT&RUN and ATAC seq experiments were performed in biological duplicates for each genotype and tissue. minION long-read sequencing was performed in singletons for each genotype because a single sequencing read across the transgene is sufficient to confirm the structure of the allele. CHiC was performed as singleton experiment on a pool of tissues for 6 or 7 embryos because the observed change is so broad and robust we decided that replication would not add meaningful data to the interpretation. High throughput sequencing experiments were performed in biological duplicates because they were concordant, and meaning will not be gained from more samples, and don't justify the cost of reproducing a third replicate. Capture Hi-C was performed as a single replicate

	because the result was clear, and we are not trying to estimate variation across individuals.
Data exclusions	Some embryos were excluded from the analysis of CRISPR induced mutagenesis experiments (Figure 4A-B and Figure 5). In some embryos we could not detect the expected wild type or mutation structure by genotyping PCR. Probably these embryos carried complex reorganizations of the genome around the cut sites. So we excluded embryos from the analysis if we could not confirm the positive presence of the expected mutation.
Replication	For all WISH, at least 3 biological replicates were evaluated (3 embryos), which is a commonly accepted sample size for such experiments. A sample size of 3 can demonstrate that a change in gene expression is consistent across genotypes, while balancing for animal usage considerations. For the figures, one embryo was chosen as representative of the set.<br><br>For CUT&RUN and ATAC (see the ChIP-seq section details below) biological replicates were performed. For each replicate, tissues from 3 to 5 embryos were pooled for each genotype and tissue. Separate pools of cells (from different embryos) were used for each ATAC and CUT&RUN experiment. For CHiC-seq, the tissue was pooled from 6 or 7 embryos of the same genotype in order to produce enough material for experimentation. As these experiments result from pools of cells from several embryos, the resulting data is an average of measurements across many samples.<br><br>The minION sequencing was performed as a singleton experiment for each genotype.<br>In the control embryos for Figure S4-3, for each construct inserted into the TDOM site (Del 3x13 and HBB:lacZ), a single clone of embryonic stem cells was used for implantation in to embryos. Multiple embryos were collected for each clone and stained.<br><br>All attempts at replication were successful.<br><br>For Figure 1C we report the number of embryos that stain in the results section.<br>For the Il1 TDOM lacZ stains in Figure 2 we stained dozens of embryos at different stages and in the heterozygous and homozygous genetic states.<br><br>For the embryos generated by random transgenesis (Figure 4), we performed 2 to 4 rounds of injection per construct. In total, embryos were harvested from 7 to 15 females per construct. Images of all the embryos presenting a lacZ staining are provided in Supplementary Figure 4-2. They were obtained from at least 3 different females per construct.<br><br>We also provide images of all stained embryos carrying large deletions (Figure 5 and Supplementary Figure 5).
Randomization	No experiments required randomization as experimental groups are based on genotypes.
Blinding	Blinding is not relevant in this study because experimental groups are based on genotypes.

Reporting for specific materials, systems and methods

We require information from authors about some types of materials, experimental systems and methods used in many studies. Here, indicate whether each material, system or method listed is relevant to your study. If you are not sure if a list item applies to your research, read the appropriate section before selecting a response.

Materials & experimental systems

n/a	Involved in the study
☐	☒ Antibodies
☐	☒ Eukaryotic cell lines
☒	☐ Palaeontology and archaeology
☐	☒ Animals and other organisms
☒	☐ Human research participants
☒	☐ Clinical data
☒	☐ Dual use research of concern

Methods

n/a	Involved in the study
☐	☒ ChIP-seq
☒	☐ Flow cytometry
☒	☐ MRI-based neuroimaging

Antibodies

Antibodies used	anti-HOXD13 Abcam Ab19866 Lot#GR3290844-1, anti-HOXA13 Abcam ab106503 Lot#GR3243694-1
Validation	anti-HOXD13 antibody was previously validated (Bolt et al., 2021, Nature Comm. Mesomelic dysplasias associated with the HOXD locus are caused by regulatory reallocations). anti-HOXA13 was validated by the manufacturer with Western Blot and Immunohistochemistry. We also validated the antibody in-house by using it in CUT&RUN experiment on E12.5 distal forelimbs that are homozygous for a Hoxa13 loss-of-function allele.

Eukaryotic cell lines

Policy information about cell lines

Cell line source(s)	Male G4 mouse ESCs (Jorge et al., 2007)

Authentication	Genetically modified pluripotent ESCs were tested by the production of embryos through tetraploid aggregations (Artus and Hadjantonakis 2011) and then genotyping confirmed the presence of the desired mutations in the cells
Mycoplasma contamination	All cell lines tested negative for mycoplasma contamination.
Commonly misidentified lines (See ICLAC register)	No commonly mis-identied cell lines were used in this study

Animals and other organisms

Policy information about studies involving animals; ARRIVE guidelines recommended for reporting animal research

Laboratory animals	Mus musculus, all animals are kept in a continous back cross with BL6 X CBA F1 hybrids. Sex of the animals was not considered in this analysis. All embryos used for WISH were at E12.5. CHi-C-seq, CUT&RUN-seq, ATAC-seq, were performed on E12.5 embryos. Mice were housed in the University of Geneva Sciences III animalarie with light 07:00-19:00 in the summer, and 06:00-18:00 in winter with ambient temperatures maintained between 22-23ºC and 45-55% humidity, the air is renewed 17-times per hour.<br>Embryos used in Supplementary Figure 4-3 were generated from male G4 mouse ESCs (Jorge et al., 2007) via tetraploid complementation (Artus and Hadjantonakis 2011). Donor tetraploid embryos were provided from in vitro fertilisation using C57Bl6J x B6D2F1 backgrounds. Aggregated embryos were transferred into CD1 foster females and micro-dissected at embryonic stage E12.5. C57Bl6, B6DF1, and CD1 mice were purchased from Janvier Labs, and used for experiments at approximately four months of age.
Wild animals	No wild animals were used in this study.
Field-collected samples	No field-collected samples were used in this study.
Ethics oversight	All experiments were performed in agreement with the Swiss Law on Animal Protection (LPA) under license numbers GE 81/14 and VD2306.2 (to D. Duboule).

Note that full information on the approval of the study protocol must also be provided in the manuscript.

ChIP-seq

Data deposition

☒ Confirm that both raw and final processed data have been deposited in a public database such as GEO.

☒ Confirm that you have deposited or provided access to graph files (e.g. BED files) for the called peaks.

Data access links<br>*May remain private before publication.*	All raw and processed datasets are available in the Gene Expression Omnibus (GEO) repository under accession number GSE194114 (secure token: yvixgaugznepdmd). All scripts necessary to reproduce figures from raw data are available at https://github.com/lldelisle/scripts/. All data will be publicly available upon publication.
Files in database submission	ChIP/SRR3168462_wt_E12_PFL_H3K27me3.bw<br>ChIP/SRR3168462_wt_E12_PFL_H3K27me3.fastq.gz<br>ChIP/SRR3168462_wt_E12_PFL_H3K27me3.narrowPeak.gz<br>ChIP/SRR3168464_wt_E12_DFL_H3K27me3.bw<br>ChIP/SRR3168464_wt_E12_DFL_H3K27me3.fastq.gz<br>ChIP/SRR3168464_wt_E12_DFL_H3K27me3.narrowPeak.gz<br>ChIP/SRR3498934_wt_E11_FL_HOXA13.bw<br>ChIP/SRR3498934_wt_E11_FL_HOXA13.narrowPeak.gz<br>ChIP/SRR3498935_wt_E11_FL_HOXD13.bw<br>ChIP/SRR3498935_wt_E11_FL_HOXD13.narrowPeak.gz<br>ChIP/SRR5855214_wt_E12_DFL_H3K27ac.bw<br>ChIP/SRR5855214_wt_E12_DFL_H3K27ac.fastq.gz<br>ChIP/SRR5855214_wt_E12_DFL_H3K27ac.narrowPeak.gz<br>ChIP/SRR5855215_wt_E12_PFL_H3K27ac.bw<br>ChIP/SRR5855215_wt_E12_PFL_H3K27ac.fastq.gz<br>ChIP/SRR5855215_wt_E12_PFL_H3K27ac.narrowPeak.gz<br>ChIP/SRR5855220_wt_E12_DFL_CTCF.bw<br>ChIP/SRR5855220_wt_E12_DFL_CTCF.fastq.gz<br>ChIP/SRR5855220_wt_E12_DFL_CTCF.narrowPeak.gz<br>ChIP/SRR5855221_wt_E12_PFL_CTCF.bw<br>ChIP/SRR5855221_wt_E12_PFL_CTCF.fastq.gz<br>ChIP/SRR5855221_wt_E12_PFL_CTCF.narrowPeak.gz<br>CUTandRUN/542_E11.5_DFL_HOXD13_542mapping.bw<br>CUTandRUN/542_E11.5_DFL_HOXD13_542mapping.narrowPeak.gz<br>CUTandRUN/542_E11.5_DFL_HOXD13_R1.fastq.gz<br>CUTandRUN/542_E11.5_DFL_HOXD13_R2.fastq.gz<br>CUTandRUN/542_E12.5_DFL_HOXA13_rep1_542mapping.bw<br>CUTandRUN/542_E12.5_DFL_HOXA13_rep1_542mapping.narrowPeak.gz<br>CUTandRUN/542_E12.5_DFL_HOXA13_rep1_R1.fastq.gz

CUTandRUN/542_E12.5_DFL_HOXA13_rep1_R2.fastq.gz
CUTandRUN/542_E12.5_DFL_HOXA13_rep2_542mapping.bw
CUTandRUN/542_E12.5_DFL_HOXA13_rep2_542mapping.narrowPeak.gz
CUTandRUN/542_E12.5_DFL_HOXA13_rep2_R1.fastq.gz
CUTandRUN/542_E12.5_DFL_HOXA13_rep2_R2.fastq.gz
CUTandRUN/wt_E12.5_DFL_HOXA13_rep1_mm10mapping.bw
CUTandRUN/wt_E12.5_DFL_HOXA13_rep1_mm10mapping.narrowPeak.gz
CUTandRUN/wt_E12.5_DFL_HOXA13_rep1_R1.fastq.gz
CUTandRUN/wt_E12.5_DFL_HOXA13_rep1_R2.fastq.gz
CUTandRUN/wt_E12.5_DFL_HOXA13_rep2_mm10mapping.bw
CUTandRUN/wt_E12.5_DFL_HOXA13_rep2_mm10mapping.narrowPeak.gz
CUTandRUN/wt_E12.5_DFL_HOXA13_rep2_R1.fastq.gz
CUTandRUN/wt_E12.5_DFL_HOXA13_rep2_R2.fastq.gz
CUTandRUN/wt_E12.5_DFL_HOXA13_rep3_mm10mapping.bw
CUTandRUN/wt_E12.5_DFL_HOXA13_rep3_mm10mapping.narrowPeak.gz
CUTandRUN/wt_E12.5_DFL_HOXA13_rep3_R1.fastq.gz
CUTandRUN/wt_E12.5_DFL_HOXA13_rep3_R2.fastq.gz
CUTandRUN/wt_E12.5_DFL_HOXD13_rep1_mm10mapping.bw
CUTandRUN/wt_E12.5_DFL_HOXD13_rep1_mm10mapping.narrowPeak.gz
CUTandRUN/wt_E12.5_DFL_HOXD13_rep1_R1.fastq.gz
CUTandRUN/wt_E12.5_DFL_HOXD13_rep1_R2.fastq.gz
CUTandRUN/wt_E12.5_DFL_HOXD13_rep2_mm10mapping.bw
CUTandRUN/wt_E12.5_DFL_HOXD13_rep2_mm10mapping.narrowPeak.gz
CUTandRUN/wt_E12.5_DFL_HOXD13_rep2_R1.fastq.gz
CUTandRUN/wt_E12.5_DFL_HOXD13_rep2_R2.fastq.gz
CUTandRUN/wt_E12.5_DFL_HOXD13_rep3_1.fastq.gz
CUTandRUN/wt_E12.5_DFL_HOXD13_rep3_2.fastq.gz
CUTandRUN/wt_E12.5_DFL_HOXD13_rep3_mm10mapping.bw
CUTandRUN/wt_E12.5_DFL_HOXD13_rep3_mm10mapping.narrowPeak.gz

Genome browser session
(e.g. UCSC)

no longer applicable

Methodology

Replicates

CUT&RUN and ATAC-seq experiments were performed on biological duplicates. CHiC was performed on a singleton which was composed of a pool of tissues.

Sequencing depth

For ChIP
SRR3168462_wt_E12_PFL_H3K27me3: sequenced to 232.296 million reads at 100bp single-read
SRR3168464_wt_E12_DFL_H3K27me3: sequenced to 129.645 million reads at 100bp single-read
SRR5855214_wt_E12_DFL_H3K27ac: sequenced to 804.795 million reads at 50bp single-read
SRR5855215_wt_E12_PFL_H3K27ac: sequenced to 848.243 million reads at 50bp single-read
SRR5855220_wt_E12_DFL_CTCF: sequenced to 724.561 million reads at 50bp single-read
SRR5855221_wt_E12_PFL_CTCF: sequenced to 757.732 million reads at 50bp single-read
SRR3498934_wt_E11_FL_HOXA13: sequenced to 71.8 million pairs at 50bp both pairs
SRR3498935_wt_E11_FL_HOXD13: sequenced to 115.1 million pairs at 50bp both pairs

For CUT&RUN:
542_E11.5_DFL_HOXD13: sequenced to 165.438 million pairs at 93bp both pairs
542_E12.5_DFL_HOXA13_rep1: sequenced to 163.919 million pairs at 75bp both pairs
542_E12.5_DFL_HOXA13_rep2: sequenced to 118.001 million pairs at 75bp both pairs
wt_E12.5_DFL_HOXA13_rep1: sequenced to 145.825 million pairs at 75bp both pairs
wt_E12.5_DFL_HOXA13_rep2: sequenced to 128.81 million pairs at 75bp both pairs
wt_E12.5_DFL_HOXA13_rep3: sequenced to 120.21 million pairs at 84bp both pairs
wt_E12.5_DFL_HOXD13_rep1: sequenced to 105.512 million pairs at 75bp both pairs
wt_E12.5_DFL_HOXD13_rep2: sequenced to 165.421 million pairs at 75bp both pairs
wt_E12.5_DFL_HOXD13_rep3: sequenced to 99.9152 million pairs at 84bp both pairs

Antibodies

anti-HOXD13 Abcam Ab19866 Lot#GR3290844-1, anti-HOXA13 Lot#GR3243694-1

Peak calling parameters

ChIP PE:
```
# cutadapt version 1.16
cutadapt  -j ${GALAXY_SLOTS:-1}    -a 'TrueSeq'='GATCGGAAGAGCACACGTCTGAACTCCAGTCAC'        -A
'TruSeq'='GATCGGAAGAGCGTCGTGTAGGGAAAGAGTGTAGATCTCGGTGGTCGCCGTATCATT'      --output='out1.fq.gz' --paired-
output='out2.fq.gz'  --error-rate=0.1 --times=1 --overlap=3        --minimum-length=15 --pair-filter=any   --quality-cutoff=30
'ChIP_R1.fq.gz' 'ChIP_R2.fq.gz'  > report.txt

# bowtie2 version 2.4.1
bowtie2  -p ${GALAXY_SLOTS:-4}  -x '/data/galaxy/galaxy/var/tool-data/mm10_UCSC/bowtie2_index/mm10_UCSC/mm10_UCSC'   -1
```

```
'out1.fq.gz' -2 'out2.fq.gz'            2> 'mapping stats.txt'  | samtools sort --no-PG -@${GALAXY_SLOTS:-2} -T "${TMPDIR:-.}" -O bam -o
'bowtie2 output (BAM).bam'

# samtools version 1.8
samtools view -o 'filtered BAM.bam' -h   -b  -q 30 -f 0x2 'bowtie2 output (BAM).bam'

# macs2 version macs2 2.1.1.20160309
macs2 callpeak   -t 'filtered BAM.bam'  --name SRR3498934    --format BAMPE   --gsize '1870000000'         --call-summits  --keep-dup
'1'   --bdg  --qvalue '0.05'  --mfold '5' '50'  --bw '300'  2>&1 > macs2_stderr

ChIP SR:
# cutadapt version 1.16
cutadapt  -j ${GALAXY_SLOTS:-1}     -a 'TrueSeq'='GATCGGAAGAGCACACGTCTGAACTCCAGTCAC'      --output='out1.fq.gz'  --error-
rate=0.1 --times=1 --overlap=3        --minimum-length=15   --quality-cutoff=30   'SRR5855214.fq.gz'  > report.txt

# bowtie2 version 2.4.1
bowtie2  -p ${GALAXY_SLOTS:-4}  -x '/data/galaxy/galaxy/var/tool-data/mm10_UCSC/bowtie2_index/mm10_UCSC/mm10_UCSC'   -U
'out1.fq.gz'              2> 'mapping stats.txt'  | samtools sort --no-PG -@${GALAXY_SLOTS:-2} -T "${TMPDIR:-.}" -O bam -o 'bowtie2
output (BAM).bam'

# samtools version 1.8
samtools view -o 'filtered BAM.bam' -h   -b  -q 30 'bowtie2 output (BAM).bam'

# macs2 version macs2 2.1.1.20160309
macs2 callpeak   -t 'filtered BAM.bam'  --name SRR5855214    --format BAM   --gsize '1870000000'         --call-summits  --keep-dup '1'
--bdg  --qvalue '0.05'  --mfold '5' '50'  --bw '300'  2>&1 > macs2_stderr

CUT&RUN:
# cutadapt version 1.16
cutadapt  -j ${GALAXY_SLOTS:-1}     -a 'Truseq R1'='GATCGGAAGAGCACACGTCTGAACTCCAGTCAC'       -A 'TruSeq
R2'='GATCGGAAGAGCGTCGTGTAGGGAAAGAGTGTAGATCTCGGTGGTCGCCGTATCATT'      --output='out1.fq.gz' --paired-
output='out2.fq.gz'  --error-rate=0.1 --times=1 --overlap=3        --minimum-length=15 --pair-filter=any   --quality-cutoff=30
'CUTandRUN_R1.fq.gz' 'CUTandRUN_R2.fq.gz'  > report.txt

# bowtie2 version 2.4.1
bowtie2  -p ${GALAXY_SLOTS:-4}  -x 'genome'   -1 'input_f.fastq.gz' -2 'input_r.fastq.gz' -I 0 -X 1000 --fr --no-mixed --no-discordant --
dovetail              --very-sensitive   2> 'mapping stats.txt'  | samtools sort --no-PG -@${GALAXY_SLOTS:-2} -T "${TMPDIR:-.}" -O bam -o
'bowtie2 output (BAM).bam'

# samtools version 1.8
samtools view -o 'filtered BAM.bam' -h   -b  -q 30 -f 0x2 input.bam

# picard version 2.18.2
_JAVA_OPTIONS=${_JAVA_OPTIONS:-'-Xmx2048m -Xms256m'}
export _JAVA_OPTIONS
picard MarkDuplicates  INPUT='filtered BAM.bam' OUTPUT='BAM filtered rmDup.bam'  METRICS_FILE='MarkDuplicates metrics.txt'
REMOVE_DUPLICATES='false' ASSUME_SORTED='true'  DUPLICATE_SCORING_STRATEGY='SUM_OF_BASE_QUALITIES'
OPTICAL_DUPLICATE_PIXEL_DISTANCE='100'   VALIDATION_STRINGENCY='LENIENT' QUIET=true VERBOSITY=ERROR  `if [ -n
"$TMPDIR" ] ; then echo 'TMP_DIR=$TMPDIR' ; else if [ -n "$TEMP" ] ; then echo 'TMP_DIR=$TEMP' ; fi ; fi`

# bedtools version 2.29.2
bedtools bamtobed    -i 'BAM filtered rmDup.bam' > 'BED filtered rmDup.bed'

# macs2 version 2.1.1.20160309
macs2 callpeak   -t 'BED filtered rmDup.bed'  --name CUTandRUN    --format BED   --gsize '1870000000'         --call-summits  --keep-
dup 'all'   --bdg  --qvalue '0.05'  --nomodel --extsize '200' --shift '-100'  2>&1 > macs2_stderr
```

Data quality

All ChIP were previously published so we don't assess their quality here.
CUT&RUN for HOXA13/HOXD13 get between 24'000 and 118'000 peaks with FDR less than 5% and between 4'700 and 35'700 with FDR less than 5% and fold-change above 5.

Software

All scripts necessary to reproduce figures from raw data are available at https://github.com/lldelisle/scriptsForBoltEtAl2022.
